# Medical Cannabis Activity Against Inflammation: Active Compounds and Modes of Action

**DOI:** 10.3389/fphar.2022.908198

**Published:** 2022-05-09

**Authors:** Seegehalli M. Anil, Hadar Peeri, Hinanit Koltai

**Affiliations:** Institute of Plant Science, Agriculture Research Organization, Volcani Center, Rishon LeZion, Israel

**Keywords:** inflammation, medicinal cannabis, phytocannabinoids, *Δ* 9-tetrahydrocannabinol (THC), cannabidiol (CBD), cannabigerol (CBG)

## Abstract

Inflammation often develops from acute, chronic, or auto-inflammatory disorders that can lead to compromised organ function. Cannabis (*Cannabis sativa*) has been used to treat inflammation for millennia, but its use in modern medicine is hampered by a lack of scientific knowledge. Previous studies report that cannabis extracts and inflorescence inhibit inflammatory responses *in vitro* and in pre-clinical and clinical trials. The endocannabinoid system (ECS) is a modulator of immune system activity, and dysregulation of this system is involved in various chronic inflammations. This system includes cannabinoid receptor types 1 and 2 (CB1 and CB2), arachidonic acid-derived endocannabinoids, and enzymes involved in endocannabinoid metabolism. Cannabis produces a large number of phytocannabinoids and numerous other biomolecules such as terpenes and flavonoids. In multiple experimental models, both *in vitro* and *in vivo*, several phytocannabinoids, including Δ9-tetrahydrocannabinol (THC), cannabidiol (CBD) and cannabigerol (CBG), exhibit activity against inflammation. These phytocannabinoids may bind to ECS and/or other receptors and ameliorate various inflammatory-related diseases by activating several signaling pathways. Synergy between phytocannabinoids, as well as between phytocannabinoids and terpenes, has been demonstrated. Cannabis activity can be improved by selecting the most active plant ingredients (API) while eliminating parts of the whole extract. Moreover, in the future cannabis components might be combined with pharmaceutical drugs to reduce inflammation.

## Introduction

The immune system consists of both adaptive and innate immunity. Innate immunity is the rapid and non-specific response to pathogens mediated by myeloid cells and natural killer (NK) cells. On the other hand, adaptive immunity is a slower but specific response that generates immunological memory, involving the activation of B and T lymphocytes (Netea et al., 2019). During normal inflammation, innate immunity is activated within minutes to hours as a first line of defense against pathogen infection, followed by the elimination of the threats carried out by both the innate and the adaptive immune responses (Netea et al., 2019). Ending inflammation and returning to homeostasis is a process known as resolution. However, failure to remove the inciting stimulus efficiently can lead to the development of chronic inflammation and progression of tissue damage (Feehan and Gilroy, 2019). This kind of chronic, unresolved inflammation contributes significantly to various pathogeneses, including that of asthma (Pothen et al., 2015), COVID-19 (Effenberger et al., 2021), atherosclerosis (Galkina and Ley, 2009), chronic obstructive pulmonary disease (Sevenoaks and Stockley, 2006), inflammatory bowel disease (Actis et al., 2019), neurodegenerative disease (Perry, 2004), multiple sclerosis (Sá, 2012) and rheumatoid arthritis (Masoumi et al., 2021).

Cannabis (*Cannabis sativa*) has been used as medicine for the treatment of inflammation for millennia, but its use in modern medicine has been hampered by a lack of scientific knowledge (Ryz et al., 2017). Previous studies reported that cannabis extracts and inflorescence inhibited inflammatory responses *in vitro* and in pre-clinical and clinical studies. For example, a high-CBD cannabis ethanolic extract reduced the release of skin inflammation mediators in keratinocytes (Sangiovanni et al., 2019). Similarly, a study on a mouse model of colitis showed that oral or intraperitoneally treatment with high-CBD cannabis extract led to a reduction in intestinal inflammation and hypermotility, in contrast to pure CBD treatment at matched doses (Pagano et al., 2016). Moreover, two clinical trials on patients with Crohn’s disease reported that daily treatment with THC-rich cannabis inflorescence had beneficial effects against the disease symptoms with no significant side effects and reduced the need for other medications (Naftali et al., 2011; Naftali et al., 2013). In another clinical trial, daily cannabis treatment was associated with lower levels of pro-inflammatory biomarkers in cerebral fluid (CSF) of HIV patients (Watson et al., 2021).

Great efforts have been made to suppress chronic inflammation. Cannabis and its compounds were shown to have anti-inflammatory activity (see Appendix A for methodology), but to exploit the full potential of cannabis it is important to define the active molecules and understand the cellular and molecular mechanisms that underlie its anti-inflammatory activity.

## A Brief Description of the Cornerstones of Inflammation

**FIGURE 1:**
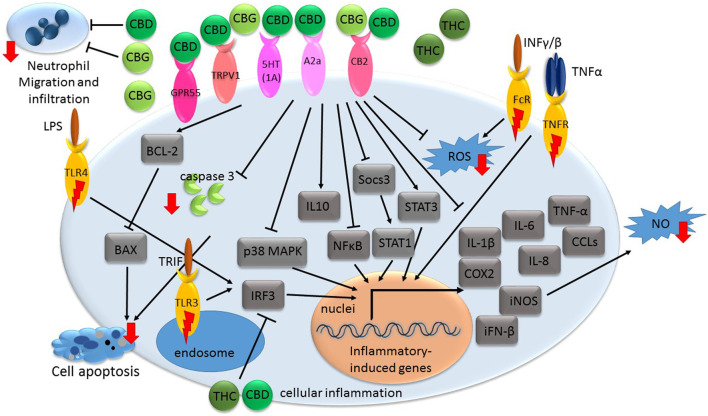
A general illustration of some of the signaling pathways suggested being associated with phytocannabinoid-mediated inflammation suppression. Receptors with inflammatory-inducing activity are marked with a red lightning bolt. Other receptors interact with phytocannabinoids to convey anti-inflammatory responses. Genes or proteins are designated in rectangular boxes. Red arrows denote reduction in biological processes or components following cannabinoid treatments. CBD-cannabidiol; CBG-cannabigerol; THC- *Δ* ^9^-tetrahydrocannabinol; CB- cannabinoid receptor; GPR- G protein-coupled receptor; TRPV- transient receptor potential vanilloid; A2a-adenosine receptor; iFN- interferon; TNF- tumor necrosis factor; CCL- C-C motif chemokine; IL-interleukin; COX-cyclooxygenase; iNOS- nitric oxide synthase; ROS- reactive oxygen species; NO- nitric oxide; MAPK- mitogen-activated protein kinase; LPS- bacterial lipopolysaccharide; NFκB- nuclear factor kappa B; IRF3- regulatory factor 3; FcR- Fc receptor; TNFR- TNF receptor; INF- interferon; 5HT(1A)- serotonin receptor; TLR-toll-like receptor; TRIF- Toll-Interleukin-1 Receptor (TIR)-domain-containing adaptor-inducing interferon-*β*.

Monocytes are the major starting entities of inflammation. Once released from bone marrow, monocytes migrate through the blood into various tissues and undergo the tissue-specific maturation required to become inflammatory macrophages that respond to infection, injury, or damage. The various sub-populations of activated macrophages may differ in morphology, release of inflammatory mediators and functional properties, but in inflammation they have three major functions: phagocytosis, antigen presentation and immunomodulation (Fujiwara and Kobayashi, 2005). The process of inflammation is orchestrated via inflammatory mediators. Pro-inflammatory cytokines, such as tumor necrosis factor alpha (TNF-α) and interleukin (IL)-1β are released from activated macrophages in response to infection (Figure 1; Abdulkhaleq et al., 2018). TNF-α and IL-1β act through specific cell membrane-bound receptors and participate in the recruitment of polymorphonuclear neutrophils (PMNs) into the site of infection and their activation (Hackel et al., 2021).

TNF-α facilitates the release of other pro-inflammatory cytokines from immune effector cells, including interferon alpha (IFN-*α*), interferon gamma (IFN-*γ*), IL-1β, IL-6, IL-8, Transforming growth factor beta (TGF-*β*) and chemokines (Silva et al., 2019). Further, in cases of enhanced inflammation, when the cell is stimulated, typically by bacterial lipopolysaccharide (LPS) or pro-inflammatory cytokines, there is induction of inducible nitric oxide synthase (iNOS). An increase in iNOS levels generates significant amounts of nitric oxide (NO) radicals or cyclooxygenase 2 (COX2); COX2 catalyzes the conversion of arachidonic acid to prostaglandins (PGs), prostacyclin and thromboxane A_2_ (Salvemini et al., 2013; Cinelli et al., 2020).

The signal transduction of inflammatory responses involves several signaling pathways including mitogen-activated protein kinase (MAPK), toll-like receptor (TLR), Janus kinase/signal transducers and activators of transcription (JAK-STAT), and nuclear factor kappa B (NFκB) pathways (Figure 1; Zhao et al., 2021). The activation of these pathways involves a series of phosphorylation events leading to the induction of various anti-apoptotic target genes and the expression of cytokines, chemokines, and adhesion molecules (Taniguchi and Karin, 2018; Fitzgerald and Kagan, 2020). Moreover, during inflammatory processes, reactive oxygen species (ROS) are commonly multiplied and can contribute to host cell and organ damage. Further, intracellular redox changes induced by ROS augment NF-κB activation through the phosphorylation and degradation of IκB by increasing IkB kinase *ß* (IKK) or Akt kinase activity (Haddad, 2002).

Resolution of inflammation may involve increased production of IL-10, among others. IL-10 is an anti-inflammatory cytokine, which inhibits the release of lipid mediators and pro-inflammatory cytokines (e.g., IL-1β, IL-6, and TNF-α; Figure 1; Panigrahy et al., 2021).

## The Endocannabinoid System and Inflammation

The endocannabinoid system (ECS) is a modulator of multiple physiological activities, including in the nervous, endocrine, immune, blood circulation, gastrointestinal tract and reproductive systems (Di Marzo et al., 1998). Accordingly, dysregulation of the ECS is involved with various pathological conditions, including inflammation among others (Di Marzo and Piscitelli, 2015; Hillard, 2018), whereas therapeutic modulation of ECS activity has beneficial effects on various medical conditions, including those associated with inflammation (Ambrose and Simmons, 2019; Giacobbe et al., 2021). ECS is involved in both innate and adaptive immunity and in several chronic inflammatory diseases (Chiurchiù et al., 2015). ECS includes cannabinoid receptors types 1 and 2 (CB1 and CB2, respectively) and multiple other receptors such as the peroxisome proliferator-activated receptors (PPARs) and ion channels (e.g., the transient receptor potential ankyrin [TRPA] family and the transient receptor potential vanilloid [TRPV] family) (Biringer, 2021). Also included in the ECS are the receptors’ ligand, arachidonic acid derived endocannabinoids, and enzymes for endocannabinoid metabolism (Di Marzo et al., 1998).

Most immune cells express endocannabinoids, the enzymes regulating their biosynthesis and degradation, and endocannabinoid receptors (Chiurchiù et al., 2015). Both CB1 and CB2 are expressed in immune cells, with CB2 being expressed 10–100 times higher than CB1 in these cells (Jean-Gilles et al., 2010; Rahaman and Ganguly, 2021). Moreover, CB receptor activation regulates anti-inflammatory responses. For example, activation of CB2 receptors by its agonist inhibited the release of the pro-inflammatory cytokine IL-12 and IL-23 and enhanced the release of the anti-inflammatory cytokine IL-10 from cultured activated macrophages. This study suggested that the inhibitory effect of CB2 on IL-12 production was mediated by ERK1/2-MAPK (Correa et al., 2009).

In another example, a CB2 receptor agonist reduced in human peripheral blood mononuclear cells LPS-induced ERK1/2 and NF-kB-p65 phosphorylation and release of the pro-inflammatory cytokines TNF-α, IL-1β, IL-6 and IL-8 (Capozzi et al., 2021). A selective/inverse agonist of CB2 induced the differentiation of Th0 cells into regulatory T cells (Treg) cell phenotypes in a naïve CD4^+^ T lymphocyte population isolated from a mouse spleen. The Treg phenotype is important for suppressing immune response by inhibiting T cell proliferation and cytokine production. The Treg phenotype was induced via P38 phosphorylation and STAT5A activation and was characterized by the expression of FoxP3, TGF-β and IL-10. Accordingly, treatment with this CB2 selective/inverse agonist reduced colitis severity *in vivo* (Gentili et al., 2019).

## Cannabis Biomolecules

Cannabis produces a large number of phytocannabinoids (Hanuš et al., 2016). Phytocannabinoids are aromatic oxygenated hydrocarbons, derived from meroterpenoids with a resorcinyl core structure with isoprenyl, alkyl or aralkyl substitutions. The characteristic alkyl side chain typically contains an odd number of carbon atoms (Hanuš et al., 2016; Gülck and Møller, 2020). They are produced in the plant in their acid form and are decarboxylated to the active form (Gülck and Møller, 2020). Among the phytocannabinoids, Δ^9^-trans-tetrahydrocannabinols (Δ^9^-THCs) and cannabidiols (CBDs) are the most abundant (Table 1). Cannabigerol (CBG) in its acid form (CBGA) serves as a core intermediate that diverges to provide the phytocannabinolic acids (Table 1; Hanuš et al., 2016; Tahir et al., 2021).

**TABLE 1:** Representative structures of three major phytocannabinoids.

Phytocannabinoid	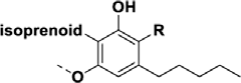
CBD	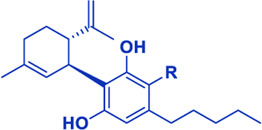
CBG	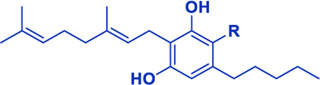
*Δ* ^9^-THC	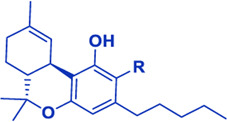

Abbreviations: CBD, cannabidiol; CBG, cannabigerol; Δ^9^-THC, Δ^9^-tetrahydrocannabinol.

In addition to phytocannabinoids, cannabis produces a plethora of non-cannabinoid constituents including a vast array of terpenes as the second-largest class of cannabis constituents (El Sohly et al., 2017). Cannabis biosynthesizes flavonoids as well, among them cannflavins, which are prenylated (C5) and geranylated (C10) flavones (Bautista et al., 2021).

## Known Phytocannabinoid Activity Against Inflammation

### Cannabidiol

CBD was demonstrated in multiple experimental models, *in vitro* and *in vivo*, to exert anti-inflammatory activities and ameliorate various inflammatory-related degenerative diseases. The mechanism of this anti-inflammatory activity is, however, not completely understood. CBD treatment of hypoxic-ischemic (HI) immature brains of newborn mice was shown to significantly reduce IL-6, TNF-α, COX-2 and iNOS expression in brain slices. This activity was suggested to be mediated via CB2 and adenosine A_2A_ receptors (Figure 1; Castillo et al., 2010). Likewise, treatments of lipopolysaccharide-treated mice with a low dose of CBD decreased TNF-α production; this effect was abolished in A_2A_ receptor knockout mice and reversed with an A_2A_ adenosine receptor antagonist, supporting the notion that CBD may enhance adenosine signaling (Carrier et al., 2006). Further, in a murine model of acute lung injury, CBD, via the A_2A_ adenosine receptor, significantly reduced leukocyte migration into the lungs and reduced the levels of albumin, TNF-α, IL-6 and other chemokines in bronchoalveolar lavage fluid (Figure 1; Ribeiro et al., 2012). CBD also reduced the activity of myeloperoxidase (MOP, an index of neutrophil infiltration) in lung tissue (Ribeiro et al., 2012).

In newborn pigs with HI brain injury, CBD administration reduced inflammation and prevented the increase in brain IL-1 levels. It also prevented the decrease in the number of viable neurons and the increase of excitotoxicity and oxidative stress. This activity was suggested to be mediated via CB2 and 5HT1A receptors (Figure 1; Pazos et al., 2013). In liver filtrate from mice with acute hepatitis, CBD was shown to trigger Myeloid-derived suppressor cells (MDSCs); these cells are regulators of the immune system that suppress T cell functions. MDSCs induction by CBD was mediated through activation of TRPV1 (Figure 1). CBD also significantly reduced blood levels of IL-2, TNF-α, IFN-γ, IL-6, IL-12, IL-17, MCP-1 and C-C motif chemokine (CCL)-11 in this model (Hegde et al., 2011).

CBD is also a selective antagonist of GPR55, another G protein-coupled receptor present in human macrophages (Figure 1). Pharmacological activation of GPR55 by its selective agonist O-1602 enhanced pro-inflammatory responses in macrophages-derived foam cells associated with a reduction in IL-10 levels and induction in TNF-α levels (Lanuti et al., 2015).

CBD treatment completely inhibited TNF-α production via p38 MAPK pathway (Figure 1) in microglial cells isolated from the retinas of newborn rats treated with endotoxin or LPS for acute ocular inflammation. In addition, LPS-treated rat retinas accumulated macrophages and activated microglia, increased levels of ROS and nitrotyrosine, and activated p38 MAPK and neuronal apoptosis. Treatment with CBD blocked all these effects (El-Remessy et al., 2008).

CBD decreases the production and release of IL-1β, IL-6 and IFN-β from LPS-activated microglial cells of BV-2 mice. CBD reduced the activity of the NF-κB pathway and the levels of IL-1β and IL-6. CBD also decreased *Socs3* gene expression; Socs3 is a main negative regulator of STATs. In accordance, CBD treatment up-regulated the STAT3 transcription factor phosphorylation, needed for its activation (Figure 1; Kozela et al., 2010). However, NF-κB and STAT3 are likely to play important and in some cases, overlapping roles in pro-inflammatory and cancer processes (He & Karin, 2011). In contrast, CBD decreased the phosphorylation of the LPS-induced STAT1 transcription factor, a key player in pro-inflammatory processes that are IFN-β-dependent (Kozela et al., 2010).

### Cannabigerol

The anti-inflammatory activity of CBG is less studied than that of CBD. Yet, several studies demonstrated significant anti-inflammatory activity of CBG. For example, CBG treatment was shown to reduce nitric oxide production in macrophages via the CB2 receptor and reduce ROS formation in intestinal epithelial cells and iNOS expression (Figure 1) in the inflamed colons. Treatment with CBG also reduced oedema in colon submucosa. This treatment also reduced the colon weight/length ratio; this ratio is a reliable marker of intestinal inflammation in a murine model of colitis glands (Borrelli et al., 2013). In addition, CBG decreased dinitrobenzene sulfonic acid (DNBS)-induced neutrophil infiltration, as evaluated by MOP activity (Borrelli et al., 2013).

In a study that characterized the anti-inflammatory properties of CBG on human skin cells *in vitro*, it was demonstrated that CBG treatment reduced ROS levels in human dermal fibroblasts, better than vitamin C. CBG also protected human epidermal keratinocytes by inhibiting pro-inflammatory cytokines that were released following induction using UVA, UVB or *Cutibacterium acnes* exposure, including TNF-α, IL-1β, IL-6 and IL-8 (Figure 1; Perez et al., 2022). Furthermore, the researchers performed a single-blind clinical study on 20 healthy volunteers with sodium lauryl sulfate (SLS)-induced contact dermatitis and found that topical application of 0.1% CBG serum showed significantly lower trans-epidermal water loss (TEWL) values compared to placebo and untreated sites. Moreover, the CBG serum reduced redness and inflammation following 48 h treatment, and after 2 weeks of application, the skin condition almost returned to baseline levels of visual grade (Perez et al., 2022).

Several studies have described the neuroprotective properties of CBG against inflammation. It was demonstrated that CBG pre-treatment of cultured motor neurons not only reduced the levels of pro-inflammatory cytokines, including IL-1β, TNF-α and IFN-γ (Figure 1), but also inhibited apoptosis in LPS-stimulated macrophages, via suppression of caspase-3 and Bax expression and induction of Bcl-2 levels (Gugliandolo et al., 2018). In addition, in a study that examined the effects of CBG on Huntington’s disease pathology in 3-nitropropionate model *in vivo*, it was found that treatment with the phytocannabinoid reduced neuronal death by half and significantly attenuated the upregulation of expression of COX-2, iNOS and pro-inflammatory cytokines such as TNF-α and IL-6 (Figure 1; Valdeolivas et al., 2015).

### *Δ* ^9^-Tetrahydrocannabinol

Several experiments suggest that THC has anti-inflammatory effects. For example, topical treatment of THC on DNFB-mediated allergic contact dermatitis in mice revealed that THC effectively decreased myeloid immune cell infiltration and contact allergic ear swelling (Gaffal et al., 2013). These anti-inflammatory effects were evident in both wild-type and CB1/2 receptor-deficient mice suggesting that these activities of THC were not mediated via CB1 or CB2 receptors. In addition, THC reduced the production by epidermal keratinocytes of CCL8 and CCL2 induced by IFNγ and the production of IFNγ by T cells (Figure 1). As a result, in a CB1/2 receptor-independent way, THC limited the recruitment of myeloid immune cells *in vitro* (Gaffal et al., 2013).

Interestingly, in LPS-induced macrophages, THC (and CBD) attenuated TLR3/4 signaling in a MyD88-independent manner (Fitzpatrick et al., 2020). TLR3 signaling is mediated via a toll-interleukin-1 receptor (TIR)-domain-containing adaptor-inducing interferon-*β* (TRIF). TLR4-induced expression of regulatory factor 3 (IRF3) activation, and CXCL10 and IFN-β were repressed by the THC and/or CBD (alone or in combination) treatments. However, these phytocannabinoid treatments did not impact TNF-α/CXCL8 expression and TLR4-induced IκB-α degradation. These activities of THC and CBD were independent of the cannabinoid receptors or PPAR*γ* (Figure 1; Fitzpatrick et al., 2020). Finally, THC, dose-dependently, protected against diclofenac-induced gastric inflammation, hemorrhagic streaks and gastric ulcers in male mice, and protected against tissue damage at doses insufficient to cause common cannabinoid side effects (Kinsey and Cole, 2013).

## Synergy Between Cannabis Molecules and Formulations of Active Plant Ingredients

The synergy between phytocannabinoids (Mazuz et al., 2020; Anis et al., 2021) as well as between phytocannabinoids and terpenes (Namdar et al., 2019) has been demonstrated. Pre-clinical evidence suggests an ‘entourage effect’ might be inferred from the superior medical activities of full-spectrum cannabis extracts versus single molecules (Koltai et al., 2019). Furthermore, in some cases a “parasitage effect” might be detected, as there might also be negative molecular interactions *in vitro* (Namdar et al., 2020).

Indeed, as detailed above, phytocannabinoids are potent anti-inflammatory and immunomodulatory agents and in some cases they act via different signaling pathways. For example, although both THC and CBD decreased inflammation in LPS-activated microglial cells of a BV-2 mouse, they acted through different, although partially overlapping, mechanisms. CBD but not THC inhibited the NF-κB-dependent pathway, yet both CBD and THC regulated the IFNβ pathway activity (Kozela et al., 2010).

In order to take advantage of the synergy and to diminish negative interactions between phytomolecules, activity might be improved by selecting the most active phytomolecules while eliminating parts of the whole extract. This approach was demonstrated in the reduction of inflammation in colon cells and tissues. A THCA-rich fraction from the cannabis strain was shown to have superior activity against inflammation over the crude extract (Nallathambi et al., 2017) suggesting that the selection of active compounds may reduce the presence of inactive compounds or even those that have pro-inflammatory effects.

Moreover, in some cases, the activity of a combination of phytomolecules was found to be superior over that of a single molecule. This was demonstrated in an *in vivo* study on inflammation, where treatment with CBD combined with cannabis extract overcame the bell-shaped dose-response of purified CBD, suggesting that components found in the extract synergize with CBD to achieve the desired anti-inflammatory action (Gallily et al., 2015). In addition, a phytocannabinoid formulation showed superior activity reducing lung inflammation over the cannabis-derived fraction *in vitro*. Moreover, this particular phytocannabinoid and CBD formulation had superior activity over CBD alone (Anil et al., 2021).

## Discussion

Cannabis compounds, in some cases via the endocannabinoids system, were shown to affect some of the cornerstones of chronic inflammation. However, in light of the large number of active molecules produced by cannabis and their sometimes-synergistic interactions, there is a need to better specify cannabis-based treatments and the active compounds, while utilizing the synergy identified between cannabis phytomolecules. Thus, even if CBD or THC are considered potentially leading molecules, additional cannabis-derived compounds may be selected for improved activity.

Future approaches for improved usage of cannabis demand the development, transformation and formulation of full-spectrum cannabis extracts into active plant ingredients (APIs) to achieve higher effectivity. This might be done via careful selection of phytomolecules composition (Koltai et al., 2019; Koltai and Namdar, 2020). Notably, selecting only a few compounds for drug formulation may be compatible with modern medicine due to the potential for standardization, and careful dosing of API-based products. Importantly, once the mode of action of phytocannabinoids and that of their combination is known, APIs might be targeted towards specific mechanisms involved with inflammation.

Moreover, it might be that cannabis components can be combined with other pharmaceutical drugs to reduce inflammation. On the one hand, complementary effects might be identified due to different and perhaps complementary modes of action of cannabis compounds and pharmaceutical drugs. For example, THC was shown to reduce gastric inflammation caused by diclofenac, which may facilitate diclofenac’s effective usage against inflammation (Kinsey and Cole, 2013). On the other, CBD and THC were shown to have metabolism-dependent inhibition for Cytochrome P450 (CYP) enzymes. CYPs are responsible for drug metabolism, including detoxication and metabolic activation of xenobiotics (Yamaori et al., 2011). Hence, combined treatment with cannabis and anti-inflammatory drugs should be carefully considered.

## Abbreviations

CBD, cannabidiol; CBG, cannabigerol; Δ^9^‐THC, Δ^9^‐tetrahydrocannabinol.

## Appendix A

Methodology: To reflect on the effect of cannabis and its derived compounds on acute or chronic inflammation and the accumulating knowledge regarding cannabis active compounds and their mode of action, we have conducted a literature review using the following terms: “inflammation “, “acute inflammation”, “chronic inflammation”, “medical use of cannabis”, “therapy” “*Cannabis sativa*”, “*C. sativa*”, “cannabis”, “cannabinoids”, “terpenes”, “cannabis oil”, “adverse effects”, “endocannabinoid”, “phytocannabinoid” and “entourage effect”. The search was conducted on general and multidisciplinary research databases for peer-reviewed scientific manuscripts, including PubMed, Google Scholar, Scopus, and Web of Science.
